# The training needs for gender-sensitive care in a pediatric rehabilitation hospital: a qualitative study

**DOI:** 10.1186/s12909-020-02384-y

**Published:** 2020-11-25

**Authors:** Sally Lindsay, Kendall Kolne

**Affiliations:** Bloorview Research Institute, Holland Bloorview Kids Rehabilitation Hospital & Department of Occupational Science and Occupational Therapy, University of Toronto, Toronto, ON Canada

**Keywords:** Diversity, Gender, Healthcare, Pediatric, Thematic analysis, Training, Women

## Abstract

**Background:**

Gender is an important social determinant of health; however, clinicians often lack training in how to provide gender-sensitive care. Offering appropriate and relevant training could help to address some gender-based health inequalities. Our objective was to identify and describe the training needs for gender-sensitive care among pediatric rehabilitation healthcare providers.

**Methods:**

This study used an interpretive descriptive qualitative design to conduct interviews with 23 pediatric rehabilitation healthcare providers (19 women, 3 men, 1 transgender man), from a pediatric rehabilitation hospital in a large urban center, in Ontario, Canada from a range of disciplines. Interviews were transcribed verbatim and analyzed using an open-coding inductive thematic analysis.

**Results:**

Our analysis revealed the following themes: [1] lack of knowledge about gender-sensitive care and the need for more training; [2] content of the desired training (i.e., gender differences, effective communication and how to practice gender-sensitive care) and [3] delivery method of the training.

**Conclusions:**

Enhanced gender-sensitive training for healthcare providers is required for optimizing patient outcomes and addressing gender-based health inequalities. Educators in pediatric rehabilitation should consider developing gender-sensitive care training that is embedded within post-graduate education and also continuing education within hospitals and community care centers.

**Supplementary Information:**

The online version contains supplementary material available at 10.1186/s12909-020-02384-y.

## Background

Gender is an important social determinant of health [1]. Gender-sensitive care is a critical component of patient-centred care [2, 3] and refers to healthcare providers perceiving sex (i.e., biological aspects such as genetic makeup, hormones) and gender (i.e., roles and expectations attributed to men and women within society) differences, issues and inequalities and applying these in their clinical practice [3–6]. Gender is a social construct that varies with roles, norms and values within society and can influence health in several ways including access to resources (e.g., health, food, education), and risk-taking behaviours [4]. Research indicates that men and women often have different occupational roles that can expose them to different risks and illnesses [4]. At a population level, women often have less access to resources than do men [4] and also experience more negative health consequences [4].

Considering the role of gender within clinical practice is important because gender can influence how individuals cope with their condition in addition to affecting clinician-patient communication [2, 7]. In turn, research shows that gender-based knowledge and attitudes of healthcare providers can affect their clinical practice and quality of care. Developing an understanding of how one’s gender can influence their clinical practice is essential for avoiding and reinforcing gender stereotypes [8, 9]; however, healthcare providers often receive limited and inconsistent training related to sex and gender [3, 10].

### Gender-sensitive care

Gender-sensitive care refers to healthcare providers having the knowledge and competence in perceived existing gender norms and differences and incorporating these into their decisions and actions [2, 11]. It also refers to addressing gender inequalities and stereotypes and other attributes of care that reflect relational and other preferences (e.g., communication style, same gender clinician-patient pairing, privacy or safety needs) [3, 12, 13]. Although there is an increasing awareness of the important role of gender within pediatric health care, there are few existing gender-sensitive approaches [14–16]. It is salient to focus on gender-sensitive healthcare because clinicians’ attitudes towards gender could influence their behaviours and may bias their evaluations of themselves and their patients [2, 17]. Exploring this topic is important because many health care providers report that they lack training in gender-sensitive care approaches and this could lead to health inequalities, incorrect or delayed diagnoses and suboptimal therapies [10, 18, 19].

Gender-sensitive care involves understanding the gendered patterns and experiences of men, women and also nonbinary or gender fluid individuals [2, 7, 20]. A gender-sensitive care approach aligns with patient and family-centered care as it focuses on interacting with patients in a respectful and supportive manner, working in partnership, providing continuity of care and sharing information to make informed choices [21, 22]. Gender-sensitive approaches consider particular health care needs while addressing the causes of gender-based health inequities and ways to transform harmful gender norms, roles and relations while focusing on promoting gender equality [23, 24].

In regards to gender-sensitive training, a recent systematic review of gender-sensitive educational interventions for healthcare providers found that 29 studies involved training programs or interventions that focused on either reducing gender bias towards men and women or on addressing the specific needs of lesbian, gay, bisexual, transgender and queer (LGBTQ+) patients [3]. Thirty-seven percent of the studies in that review showed a significant improvement in gender-related knowledge, attitudes or practice after the training, which included learning sex/gender terminology, understanding gender issues and inequalities in health, stigma, discrimination and communication skills [3]. None of the gender-sensitive training interventions focused on pediatrics or rehabilitation care.

Having greater knowledge about gender-sensitive care is salient because it is associated with more positive attitudes and enhanced patient care [25, 26]. Given that gender-based health inequalities are often socially produced, they are therefore, preventable [27]. Although several recent studies have highlighted a critical need for gender-sensitivity training for healthcare providers [2, 3, 26, 28–32], little is known about the best approaches for providing such training.

### Gender and pediatric rehabilitation

Focusing on pediatric rehabilitation healthcare providers is relevant because in Canada, where this study was conducted, the majority of pediatric rehabilitation healthcare providers are women, who provide care to a population where the majority (e.g., 60% of clients at Canada’s largest pediatric rehabilitation hospital are boys) of the patients are boys/young men [33, 34]. Such gendered discrepancies are important because they could affect the development and maintenance of rapport and overall patient health outcomes [35, 36]. Further, most programs and therapy for children and youth with disabilities are designed as gender-neutral and as such, often do not consider their gender-specific needs [14, 16].

Pediatric rehabilitation is a salient area to explore because more than one in ten youth in Canada have a disability [37]. Further, gender norms and expectations start early on in childhood and shape individuals’ attitudes, experiences and behaviours which can have important longer-term outcomes [38, 39]. Previous research shows that although sex and gender can play a critical role in the incidence, clinical presentation, manifestation and health outcomes of youth with disabilities they are often viewed as non-sexual and without gender [40–42]. Additionally, youth from gender diverse groups such as LGBTQ+ have also largely been ignored within the context of pediatric rehabilitation research and the provision of care [2, 3, 14, 15, 20]. Such a lack of attention to these groups is concerning because sexual and gender minority groups often encounter significant challenges within the healthcare system including discrimination and stigma, which could affect poor health outcomes [43–47].

Although research in the area of gender-sensitive care is growing, most studies focus on the need for gender-sensitive care or exploratory research with specific adult populations (e.g., mental health, veterans’ health care, reproductive health), and focus mostly on women’s healthcare [48–52]. Of the limited research that has explored the challenges amongst providers practicing gender-sensitive care within pediatric rehabilitation they found gender differences within their clinical practice were often based on stereotypes [20]. Another challenge in providing gender-sensitive care within pediatrics is regarding privacy and ethical concerns about how and when a youth discloses their gender identity to a healthcare provider and whether or not the parents were aware of their child’s gender identity [20]. Additionally, pediatric rehabilitation care providers reported that they wanted further training in how to practice gender-sensitive care [20]. Therefore, there is a need for further exploration of the training needs amongst such health care providers.

## Methods

### Objective and design

Our objective was to identify and describe the training needs for gender-sensitive care for pediatric rehabilitation healthcare providers. An interpretive paradigm (i.e., interpretive descriptive methodology) [53] was used to guide the data collection and analysis. This method was chosen because it goes beyond describing a phenomenon and aims to explore the importance of the findings and the clinical implications [54]. The purpose of this paradigm is to understand what kinds or varieties of the phenomenon (i.e., training needs) has and what aspects it has [53]. A main assumption within this paradigm includes that it does not use pre-existing categories for sorting the data but is rather driven by the question [53]. We used a qualitative needs assessment design with structured interviews, to maximize the busy professionals time [55]. A research ethics board at a pediatric rehabilitation hospital approved this study. We followed the consolidated criteria for reporting qualitative research guidelines [56].

We used a purposive sampling strategy with the aim of having representation from various disciplinary fields within pediatric rehabilitation (e.g., medicine, nursing, occupational therapy, physiotherapy, speech language pathology). Both authors (females, with PhDs) who have training and expertise in qualitative research, interviewing and background expertise in gender-sensitive healthcare and pediatric rehabilitation, conducted the interviews.

### Context and setting

Our study was conducted in a pediatric rehabilitation hospital in a large urban center in Ontario, Canada, where children and youth can access pediatric services until they are 18 years old. Parents are often involved in the youth’s appointment at the hospital although once they are around 15–16 years of age it is common for parents to leave the room for a portion of the appointment to allow the youth to develop skills in communicating with clinicians and providing them with the opportunity to discuss sensitive topics.

### Recruitment

Our sampling strategy used invitation letters, referrals and advertisements within a pediatric rehabilitation hospital. Participants met the following inclusion criteria: a pediatric rehabilitation clinician (e.g., physician, pediatrician, nurse, social worker, occupational therapy, speech therapy, physiotherapy, psychology, therapeutic recreation etc.) who was currently practicing at the pediatric rehabilitation hospital or satellite clinic. Participants interested in the study received an information letter and consent form from the researchers. All participants provided written consent. The researchers did not have any prior relationships with the participants.

### Data collection

Audio recorded interviews were conducted by the authors and a research assistant (all females) from August to November 2019. We conducted 23 in-person interviews with pediatric rehabilitation healthcare providers (see Table 1). This sample size was considered suitable for an exploratory qualitative study to capture the breadth of issues surrounding the training needs of gender-sensitive care [57, 58]. Our interview guide (see supplemental file) was informed by a systematic review on gender-sensitive care among healthcare providers [3] and was piloted tested with a pediatric rehabilitation clinician prior to conducting the interviews. The structured interviews lasted an average length of 26 min to fit within clinicians’ schedule. We asked participants to describe their current role and types of clients they work with, the importance of gender within their practice and any needs for gender-related training.

**Table 1:** Overview of participants

Participant ID	Gender	Occupation
1	Woman	Occupational Therapist
2	Woman	Registered Nurse
3	Woman	Physio and Occupational Therapy Assistant
4	Woman	Occupational Therapist
5	Woman	Communicative Disorders Assistant
6	Woman	Occupational Therapist
7	Woman	Occupational Therapist
8	Woman	Physiotherapist
9	Woman	Communicative Disorders Assistant
10	Woman	Therapeutic Recreation Specialist
11	Woman	Assistive Technology Consultant
12	Woman	Physiotherapist
13	Woman	Occupational Therapist
14	Woman	Speech-Language Pathologist
15	Woman	Social Worker
16	Man	Pediatric Rehabilitation Clinician
17	Woman	Occupational Therapist
18	Woman	Physician
19	Woman	Physician
20	Transgender man	Pediatric rehabilitation clinician^a^
21	Man	Speech-Language Pathologist
22	Woman	Physiotherapist
23	Man	Pediatric Rehabilitation Clinician

^a^This is anonymized to protect the confidentiality of this participant

### Data analysis

All audio recorded interviews were professionally transcribed verbatim and field notes were made after each interview. We then checked the transcripts for accuracy and to ensure they were anonymized and entered into Nvivo. Then, we used an inductive thematic analysis approach to explore participants’ training needs for gender-sensitive care [59]. Within interpretive description, qualitative thematic analysis is a cyclic process of engaging, assessing, planning and evaluating to explore commonalities to gain knowledge that can be applied in a wider clinical context [54]. This approach focused on familiarizing ourselves with the data, generating initial codes, revising and then defining them and developing themes [59]. Our research question guided the analysis where we looked for patterns and codes regarding training needs for gender-sensitive care. Both authors independently read the transcripts to guide the analysis where each author developed a list of preliminary codes, categories and themes. Next, we met as a team to discuss our codes, categories and themes while comparing, contrasting and revising them (e.g., combining together similar codes to create categories). We re-read the transcripts, compared and discussed until we reached consensus in the final list of codes, and categories within our coding tree. Relevant categories were combined to generate sub-themes and themes [59]. The first author applied the codes to all of the transcripts while the second author reviewed a sample of the coded transcripts to ensure accuracy in their application. We then extracted relevant quotes representing each theme and sub-theme while considering the whole context of the interview [59]. The second author reviewed the extracted quotes to ensure that they reflected each of the themes. Our team agreed that we reached thematic saturation within our sample, both in the codes and meaning of the codes [58].

We applied several approaches to addresses the rigour and trustworthiness of our findings including prolonged engagement, peer debriefing (e.g., discussions amongst the research team) and descriptive participant accounts to illustrate the themes [60, 61]. We kept a log of key decisions made during the data analysis and followed the consolidated criteria for reporting in qualitative research checklist. We kept an audit trail of key decisions that were made during the analysis where we also reflected on our backgrounds and social locations and considered how any bias of our experience may have influenced the interpretation of the findings [61].

### Sample characteristics

Twenty-three pediatric rehabilitation healthcare providers (19 women, 3 men, 1 transgender person) were interviewed from the following professions: occupational therapy, nursing, physiotherapy, speech language pathology, therapeutic recreation, social work, medicine, and assistive technology consultant (see Table 1).

## Results

Our analysis revealed the following themes: (1) lack of knowledge about gender-sensitive care and the need for more training; (2) content of the desired training; and (3) delivery method of the training (see Table 2 for overview of themes and sub-themes).

**Table 2:** Overview of themes and sub-themes

Themes	Sub-themes	Codes
Lack of knowledge about gender-sensitive care and the need for more training	Challenges addressing gender	-No / some training -Generational knowledge gap -Comfort level within hospital -Pushback from families
Content of desired gender-sensitive care training	-Gender differences and health-related inequalities -Effective communication related to gender -How to practice gender-sensitive care within clinical practice	-Gender identities (trans, non-binary) -Checklist of key differences -Gender-related terminology and pronoun use -Gender identity discussions -Being respectful -Understanding gender issues
Delivery method of the training	-Variety of training options -Online training -Lunch and learns -Workshops -Learning from lived experience	-Flexible -Informal learning -Question and discussion -Trans and non-binary

### Lack of knowledge of gender-sensitive care and need for training

The first theme involved a lack of knowledge regarding gender-sensitive care where the majority of participants reported that their greatest challenge in addressing gender within their practice was a lack of training in gender-sensitive care. For example, a clinician said, “I don’t remember receiving any gender-specific training” (#21). A few participants received “some training in pronouns but that’s the bulk of it” (#22). Several participants remarked that this topic was important but were unsure about how to access such information and resources. For example, a clinician shared, “I sense it’s important and I don’t know where to start … any information would be appreciated” (#1).

Several clinicians described an intergenerational knowledge gap in gender-sensitive care. For example, one person mentioned “It’s new for older people who didn’t grow up with this; but for the younger generation, it’s part of their everyday language” (#9). Another participant shared, “It was just something that wasn’t part of our fabric of life and it wasn’t talked about a lot” (#15). Other clinicians agreed that health care providers “who are a little bit older haven’t had much exposure and it would be important to start with laying the groundwork … to understand how it impacts care … when you think about clinicians who are already practicing, they can be very stubborn” (#4).

#### Challenges addressing gender

A sub-theme that was related to the lack of knowledge about gender-sensitive care was regarding the challenges that participants encountered in addressing gender within their practice especially in working with young clients and their parents. For example, a participant mentioned the comfort level within the hospital to discuss gender identities was lacking. That is, a social worker said, “at the hospital here, people aren’t totally comfortable and are still feeling like they have to pass (i.e., as a binary male/female gender identity)” (#15). Additionally, a physiotherapist explained that a challenge with addressing gender was due to a “fear of push back from families” (#22). Participants described how the nature of the pediatric setting, where parents are often in the clinical visit with the child/youth, might influence any discussions they were hoping to have related to gender or gender identity.

### Content of the desired gender-sensitive care training

A second main theme involved the content of the desired gender-sensitive training. Sub-themes involved wanting more training on gender-related differences and health-related inequalities, effective communication related to gender (including gender terminology and pronouns), and how to apply gender-sensitive care within their practice.

#### Gender differences and health-related inequalities

Many participants expressed a need for more training on gender-related health inequalities within their practice. For example, a participant reported that a key challenge to addressing gender within clinical practice was “a lot of the research has been historically done on middle-aged, white men and a lot of those symptoms don’t apply … men and women have different symptoms but we are all taught the male ones. We need to learn those differences so we know when to flag them … If we are only being taught the men stuff then that is difficult” (#22). A physician described that it would be helpful to learn about the key gender-related health differences and concerns amongst the client group that she sees. To illustrate, she said,

“I’d love to receive training on gender-specific medial concerns we should be aware of … a child who has (cerebral palsy) there are certainly things they are more at risk of or complications … with their increased risk for mental health concerns.” (#18)

Meanwhile, some participants wanted more training on health issues related to gender identity and especially how to have such discussions with youth; and for client groups where this may be more predominant. For instance, a clinician shared, “70% of people with autism don’t identify as men or women heterosexual. It’s a very interesting population where it is so prominent … that information should be shared … It would be great to have autistic people come in themselves and talk about their identities” (#22). Participants told us about how it could be somewhat of a sensitive issue to discuss gender identity with their young clients because some of them might not have told their parents yet.

#### Effective communication related to gender

Another sub-theme regarding the desired content for gender-sensitive care training involved effective communication related to gender including gender terminology and pronoun use which involved. Specifically in working with pediatric clients, many participants reported feeling unprepared with how to have gender-related discussions. For example, an occupational therapist described:

“It’s a matter of feeling unequipped to do my duty as a clinician due to gender; A barrier between my understanding and being able to communicate with someone because of gender … I’d be excited for an initiative to come forward … It could address things we take for granted that may be harming our relationships with our clients.” (#17)

In particular, participants shared their concern about how to discuss gender-identity with a young person when they may still be at a stage where they are trying to figure it out themselves.

Some participants advocated for practical training regarding effective gender-related communication. For instance, a clinician explained,

“Students should experience more realistic situations. When I was on placement, things were sugar-coated … To be a successful professional you need to deal with all of those things. Sex and gender should be part of the learning experience … Practice having those conversations” (#22).

Aligned with effective communication, most clinicians wanted more training on gender-based terminology and pronoun use. For instance, one participant said, “There’s so much terminology; you are always worried about making a mistake” (#6). Others agreed, “Having a better understanding of what are the different ways that people identify their gender or nonbinary and terms people are using” (#13).

The majority of participants desired more training on pronoun use. A nurse mentioned, “It would be helpful to have some training and build knowledge on different pronouns, different ways people identify, having somebody come in and talk about what their care is like when we don’t meet their expectations of gender identity” (#2). Others agreed, “We’re all about client-centered care and that is using whatever pronoun they want to use, or being sensitive to their needs” (#13).

#### How to apply gender-sensitive care within practice

Another sub-theme regarding the content of desired gender-sensitive care training involved how to apply gender-sensitive care within clinical practice which involved having an understanding of gender issues and being respectful. For instance, a physiotherapist explained, “By having a better understanding of gender-based issues we can provide more respectful and customized care, which will lead to better health outcomes. If people feel confident and respected by their healthcare providers, then they can participate in the rehabilitation process” (#8). Another clinician, a therapeutic recreation specialist said, “It’s an area I admittedly need to learn more about and want to be prepared so I can be a respectful clinician” (#10).

Several clinicians mentioned how learning about gender-sensitive care could help to address certain uncertainties, especially with how gender is documented or discussed. To illustrate, “It’s important to have discussions about it … where you are learning about the rules and regulations for documentation and for disclosing personal health information … and understanding gendering practice too” (#4). Participants told us that having effective procedures in place is especially important in a pediatric setting because young people may not be ready to share their gender identity with their parents. Others said it was important to tie training into their overall practice: “A little bit of understanding of what it will mean for practice … being explicit just so you’re aware and sensitive … It goes back to general reflexivity in your practice of understanding the filters through which you understand and provide advice” (#17).

### Delivery method

A third key theme involved delivery method of the training. Participants described formal and informal methods of training in gender-sensitive care. In particular, our sub-themes highlight that participants wanted a variety of options including such things as online training, workshops, lunch and learn (i.e., informal) discussions, and learning from the patient’s lived experiences.

#### Variety of training options

Many participants described the importance of having multiple ongoing strategies for delivering training on this topic. For example, “This is an evolving area and more people are vocal and able to share information. It’s not a one-stop training, but ongoing updates” (#22). Another clinician said, “multiple strategies of engagement that are offered at different times so you can provide multiple ways of engagement” (#21). They described that it was critical for training to be embedded within institutional and ongoing initiatives. Another occupational therapist thought, “It’s sort of integrated through your day-to-day. You might have a client here and then not again for two months, or maybe you will have five at once. So, having that more integrated would be helpful” (#7).

Participants emphasized the importance of making this type of training mandatory. That is, “as the healthcare system is trying to become a safer, it would make it a safer space for everybody; if we all had the same level of education … having mandatory education institution-wide is very useful, because then you are catching everybody” (#21). These healthcare professionals wanted practical and interactive gender-sensitive training. For instance, one said, “You need to have hands-on training rather than just a seminar telling people about it” (#22). Another participant agreed, “some role playing could bring a more practical sense … I’m big on practical learning, not necessarily reading it or watching a lecture” (#9).

#### Online training

There was inconsistency in the findings regarding having online training to learn about gender-sensitive care where some people wanted this delivery method and others did not. For example, a physiotherapist said, “oftentimes the way we have education through online training modules; you have to get it done by a certain time. People are often rushing through and not necessarily absorbing the information and applying it” (#12). On the other hand, a clinician mentioned that having online training could be practical for learning at your own pace and could complement other training. For example, “some people want to sit in their office and do it” (#17).

#### ‘Lunch and learns’ and informal discussions

Given how busy clinicians schedules often are, many of them wanted an informal discussion and learning environment over their lunch hour (i.e., lunch and learn). For example, a participant said, “I’d rather take an hour one afternoon on a specific topic. I like lunch and learns … I’d love to have some more gender issues at work” (#5). Others described this type of training material: “I really enjoy if people put up videos of actual experiences … .it’s one thing to read it in a book. It’s another thing to see it. So, if there are summaries of best practices, guidelines, that would be helpful” (#1). Participants mentioned a benefit of this informal learning was they could share case studies within a safe learning environment. Additionally, a physiotherapist explained: “having a case study involving gender or having some sort of discussion that is a bit less formal among colleagues … could probably help to bring people to a level playing ground” (#12).

#### Workshops

Some participants wanted to learn about gender-sensitive care through a workshop format. For instance, a clinician explained, “workshops are the most helpful because of the opportunity for people to ask questions and have discussions … There’s a lot of value in learning from each other and having a safe space to ask questions” (#7). An occupational therapist mentioned, “having speakers and a panel of people who have worked in healthcare … that’s probably best for us in terms of time management … ongoing education is helpful” (#6).

#### Learning from lived experience perspective

The majority of participants wanted to hear about client’s lived experiences. To illustrate, a clinician explained, “more stories … it’s good to hear from the consumer themselves … there’s a certain comfort and authenticity when you hear from people who have gone through the journey” (#15). Another clinician explained, “It’s more learning from people’s practical experiences about what they found helpful when working with healthcare providers … learning through people’s lived experience” (#6).

Participants in our study were especially interested in learning from the perspectives of transgender or nonbinary clients. For example, an occupational therapist said, “It’s about knowing how people are identifying; what are the lived experiences of those who have gone through the healthcare system as somebody who doesn’t necessarily identify as being male or female … what your needs are and how you want to be addressed” (#13). Participants shared with us how learning about such gender identities among a pediatric population may be particularly valuable because they might encounter different challenges than adults.

## Discussion

This study addressed an important gap by exploring the training needs in gender-sensitive care among pediatric rehabilitation clinicians, which is critical for addressing gender-based health inequalities and optimizing patient outcomes [3, 62]. Our results showed that pediatric rehabilitation clinicians lacked knowledge about gender-sensitive care and wanted more training. Our findings were consistent with research showing that other types of clinicians (e.g., medicine, nursing) desired more training in this area [3, 26, 28, 63]. Insufficient knowledge about gender-sensitive care is often linked with inequities and discrimination and could negatively affect health outcomes [46, 64]. Past research highlights that applying a gender-sensitive perspective in patient-centered care requires that clinicians understand gender differences, issues, inequalities and incorporate these into strategies and actions [5, 6]. Several researchers argue that service providers who ignore the sexuality and gender identity of clients often fail to deliver person-centered care [65, 66].

It is important to highlight that one of the key issues and challenges with trying to enhance gender-sensitive care training is that it requires people to be self-aware of any potential gender biases that they may have, including an awareness of how their own gender may contribute to the treatment of patients [7, 20]. Further, when training in gender-sensitive care has been offered in other settings (e.g., medicine with adult patients) it is often optional training [3]. Some evidence suggests that it may be more effective to offer such training over time and have it integrated into clinical practice to ensure successful uptake [67, 68]. Additionally, it is important to consider that gender is a part of a larger socio-cultural context and that health care organizations themselves are gendered [2]. Although gender-sensitive training is important, it is critical to recognize that gender-based stigma, discrimination and health inequities continue to exist within society and it is critical that health care providers advocate for the needs of their patients [69]. Commitment at an organizational level is also needed to help improve the implementation and uptake of gender-sensitivity training [2, 3, 10].

Another potential challenge in implementing gender-sensitive care training, especially within a pediatric setting, is that there are privacy issues that need to be considered, particularly if youth want to discuss gender-specific issues or gender identity issues without their parents knowing [20]. Other issues that are important to consider within a pediatric rehabilitation setting include that the clinicians are typically female-dominated [20], which could affect the development of rapport and communication with boys and young men who typically comprise higher proportions of the pediatric rehabilitation clients within Canada [20]. Some research shows that communication style within a medical encounter is an important way that practice behaviours differ by gender [70].

Our results highlighted the preferred content of gender-sensitive care training involved health-related gender differences, effective communication, terminology and strategies for practicing gender-sensitive care. These findings were consistent with studies showing that gender-sensitive training for other types of clinicians included terminology, gender issues, and health inequalities [3]. Training should include the use of gender-neutral language and provide inclusive questions on intake forms (i.e., gender self-assessment, anatomical structure and sex assigned at birth) [71]. Having a medical documentation system that captures gender-diversity (e.g., name, pronoun, gender identity) could help to potentially avoid misgendering a patient and the consequent stress or stigma [72]. It is important to highlight that affirming names and pronouns within a pediatric setting may require extra care when parents are present in the child’s appointment and may not be aware of their child’s preferred gender identity [20].

Our findings highlighted the delivery method of the gender-sensitive care training that participants wanted, which included having a variety of options (e.g., online training, workshops, informal discussions, and learning from patient’s lived experience). Given the complexity of gender-sensitivity, having multi-level training while using an interactive pedagogical approach is more likely to promote gender-sensitivity [65]. Although many educators recognize a need for gender-sensitive healthcare training, of the places that offer it, it is usually an optional course within postgraduate education [3]. It may be more effective to have such training as part of a longer-term, integrated structure for optimal uptake [8, 67]. Additionally, having role models, peer support or gender champions could also play a pivotal role in educating students and healthcare professionals in gender issues [2]. Indeed, there are many implications for developing gender-sensitive care training within hospitals (e.g., continuing education), in addition to the potential for embedding training within post-graduate training programs. Future research should explore the optimal timing, content, and delivery format of gender-sensitive care training. Further studies should consider whether it is worthwhile to have tailored interventions specific to the type of healthcare provider or more of a generic or interprofessional model would be more effective. The further development of gender-sensitive care training should consider co-creating with patients and include various gender perspectives.

### Limitations and future directions

It is important to acknowledge the limitations of this study. First, we drew on only one site from a large urban center and the findings may not reflect the training needs of other healthcare centers. Future studies should explore rural and suburban areas. Second, the majority of the participants in our sample were women, which is reflective of the gender distribution of pediatric rehabilitation providers and therefore, our findings may not be reflective of other genders. Further research should explore whether certain formats of delivering gender-sensitive training can affect health outcomes.

## Conclusions

Although gender is an important social determinant of health many clinicians in pediatric rehabilitation report lacking training in how to provide gender-sensitive care. This study explored the training needs for gender-sensitive care among pediatric rehabilitation clinicians. Our qualitative study highlighted a critical need for more training in gender-sensitive care. Educators should consider providing clinicians with a variety of learning options and formats to enhance their training in this area. Specific content should aim to address effective communication, terminology and how to practice gender-sensitive care within a pediatric setting, ideally, while co-creating with patients and incorporating their lived experiences. Further research is needed to understand the optimal timing, format and delivery of such training.

## Supplementary Information

**Additional file 1.** Interview guide.

## Data Availability

The data generated during the current study are not publicly available due to privacy issues, but the anonymized data will be shared by the corresponding author upon reasonable request with approval from our research ethics board.
