# Supplementary material for: The training needs for gender-sensitive care in a pediatric rehabilitation hospital: a qualitative study
Source: BMC Med Educ. 2020 Nov 25;20:468. doi: 10.1186/s12909-020-02384-y (PMC7690145; doi:10.1186/s12909-020-02384-y)
Supplement: Supplementary file 1 — Additional file 1. Interview guide. [file 12909_2020_2384_MOESM1_ESM.docx]

**Supplemental file—Interview guide**

1. Can you tell us about your current role as a clinician or trainee?
2. Tell me about working with clients that you typically work with (type of disability; gender composition of client caseload).
   1. Probe for communication with clients and families, development of rapport
   2. Assessment (identification of traditional roles; gender roles)
3. What is your overall impression of the importance of gender issues in clinical work?
   1. In what way did gender matter? Can you give an example?
   2. Can you tell us about a time or situation you were involved in where gender mattered? (how was it handled? How did you react?)
   3. Can you give an example of providing gender-sensitive care?
   4. Do you have examples of working with male and female clients? LGBT clients?
   5. Have you noticed different issues with providing care to male and female clients? If so, can you give us an example? (probe for successes and challenges)
4. Please describe any training you received in school or continuing education activities with regards to gender-sensitive care.
   1. Probe for whole course, part of a course (optional or mandatory)
   2. What resources are available to learn more about gender-sensitive care?
5. Do you feel that gender affects your practice in pediatric rehabilitation? If so, please describe how. (probe for example)
6. Are there characteristics of your work with male clients that differ from the work

your female clients? Please describe.

1. How do you work with other health care providers within pediatrics rehabilitation to

provide gender-sensitive care?

1. Do you have any recommendations of best practices in providing gender-sensitive care

for children and youth with a disability?

1. Is there anything else you would like to add that we did not get a chance to talk about?
